# Comparison of ExPress Mini-Device Implantation Alone or Combined with Phacoemulsification for the Treatment of Open-Angle Glaucoma

**DOI:** 10.1155/2015/613280

**Published:** 2015-10-20

**Authors:** Łukasz Stawowski, Joanna Konopińska, Marta Deniziak, Emil Saeed, Renata Zalewska, Zofia Mariak

**Affiliations:** Department of Ophthalmology, Medical University of Białystok, M. Skłodowskiej-Curie 24A Street, 15-276 Białystok, Poland

## Abstract

We propose comparative assessment of the effectiveness of two surgical methods for the treatment of open-angle glaucoma: (1) ExPress mini-device implantation combined with phacoemulsification and (2) ExPress mini-device implantation alone. In this prospective study, 81 patients (88 phakic eyes) with uncontrolled open-angle glaucoma enrolled for surgery. They were assigned two groups, those with coexisting cataracts (46 eyes; P-ExPress group) and those with glaucoma alone (42 eyes; ExPress group). The follow-up period was 12.9 ± 0.4 months in P-ExPress and 12.2 ± 0.6 months in ExPress group. In both groups the following parameters were measured: best corrected visual acuity (BCVA), intraocular pressure (IOP), number of complications and necessary postoperative interventions, and number of glaucoma medications. The IOP at the end of follow-up was similar in both groups (18.8 ± 5.9 versus 18.1 ± 4.8 mmHg; *P* = 0.814). There were no statistical differences in the average number of glaucoma medications between ExPress and P-ExPress groups (0.9 ± 1.65 versus 1.3 ± 1.7; *P* = 0.419) as well as in the number of postoperative complications (26 versus 21%; *P* = 0.179 in the P-ExPress and ExPress groups, resp.). Both methods are safe and effective for the surgical treatment of open-angle glaucoma. Coexistence of cataracts does not constitute a compelling contraindication for combined surgery.

## 1. Introduction

Chronic open-angle glaucoma is caused by increased outflow resistance of aqueous humor which results in increased intraocular pressure (IOP). This can generally lead to damage of the optic nerve and eventually blindness. The aim of treatment is to reduce IOP in order to prevent optic nerve damage. Surgery is indicated when the optimal pharmacological treatment fails to control IOP or when a patient is intolerant to the prescribed medications.

The ExPress miniature shunt device was introduced in 2002 for the surgical treatment of glaucoma and as a modern alternative to traditional trabeculectomy. The device is a 2.96 mm stainless steel tube with a 400 *μ*m external diameter and 50 *μ*m internal diameter [1]. Initially, this device was implanted subconjunctivally [2–4]. However, due to a substantial number of complications [5], the surgical technique was modified and current standard practice is implantation in the anterior chamber under a scleral flap [6, 7]. The ExPress device shunts aqueous humor away from the anterior chamber through a permanently open tube and is at least as efficacious at lowering IOP as conventional trabeculectomy but produces less tissue trauma [1]. ExPress implants have been used in emergencies where trabeculectomy failed or had a high probability of failure. At present, ExPress implants are frequently the first-choice surgical procedure for open-angle glaucoma.

In older people glaucoma and cataracts often present concomitantly, and patients require treatment for both conditions. The aim of the current study was to determine if combined glaucoma (ExPress implants) and cataract surgery (phacoemulsification with intraocular lens implantation) was as efficacious as glaucoma surgery alone.

## 2. Materials and Methods

The tenets of the World Medical Association Declaration of Helsinki and the principles developed by the European Union entitled Good Clinical Practice for Trials on Medical Products in the European Community were followed in this study. The project was approved by the Bioethics Committee of the Medical University of Białystok. The participants signed a written consent form to take part in the study, which lasted at least 12 months. They were informed about the treatment methods, anticipated outcome, and potential disadvantages.

The indication for surgical treatment was uncontrolled glaucoma, in some cases with a coexisting cataract. The inclusion criteria were met by patients with primary open-angle glaucoma and secondary open-angle glaucoma, pseudoexfoliative (PEX) glaucoma and pigmentary glaucoma (PG), with unsatisfactory IOP control despite maximally tolerated topical and systemic medication. Additional inclusion criteria included the following: documented loss of visual field, significant daily IOP fluctuation, noncompliance in antiglaucoma therapy, or allergy to topical medications. The exclusion criteria were history of eye surgery, closed or narrow angle glaucoma, advanced macular degeneration, active inflammatory disease, and lack of patient consent.

The prospective comparative study involved 81 patients (88 eyes). The two groups consisted of ExPress mini-device implantation combined with phacoemulsification (P-ExPress group; *n* = 46), patients with coexisting cataract, and implantation of the ExPress implant alone (ExPress group; *n* = 42), patients with no visually significant lens opacification. Surgery was carried out according to the procedure described previously [1, 6]. All surgical procedures were performed under retrobulbar anaesthesia (2% xylocaine and 0.5% bupivacaine) by two experienced surgeons (Zofia Mariak and Renata Zalewska). In both procedures, a fornix-based conjunctiva was dissected and the sclera was exposed. A limbus-based, square-shaped (4 × 4 mm) scleral flap was dissected. Then, in the P-ExPress group, clear corneal incision for phacoemulsification was made 2.75 mm from the temple, and, with the phacochop technique using the Infiniti Vision System (Alcon Surgical, Fort Worth, TX), cataract was removed and an IOL was implanted. The same IOL was used in all patients. A mini glaucoma shunt was implanted on one hour. The scleral flap was closed with 10/0 nylon sutures (4 knotted sutures) and the conjunctiva was closed with absorbable sutures. In both groups 5-fluorouracil (50 mg/mL) was used on a standard basis and applied to the scleral wound bed for 3.5 minutes to avoid contact with the conjunctiva incision area. No complications during cataract surgery occurred.

Topical antibiotic and steroid treatment was applied after surgery. Immediately after surgery, the patients stopped taking their previously prescribed glaucoma medicines unless the expected IOP reduction was not attained, as recommended by EGS.  Table 1 shows the demographic data for the patients at the start of the study.

The patients were examined 8 times, once before surgery and 7 times postoperatively. Basic postoperative examinations were carried out on the 1st, 7th, and 30th days and extensive examinations on the 3rd, 6th, 9th, and 12th months (including visual field examination and optical coherence tomography (OCT) of the optic disc).

At the initial examination prior to surgery, the patient's health and medical history was obtained, including any past treatment and surgeries. The following parameters were evaluated: uncorrected distance visual acuity (UDVA) and best corrected visual acuity (BCVA) using a Snellen chart; intraocular pressure using the Goldmann applanation tonometer (if the difference was ≥3 mmHg between the first two measures then a third reading was taken) in accordance with the Advanced Glaucoma Intervention Study, in the morning at the same time between 8:00 and 10:00; ophthalmologic examination of the anterior and posterior eye segments; axial length of the eyeball (AXL); gonioscopy; visual field examination (Humphrey, SITA Standard 24-2); OCT of the optic disc (Topcon Medical Systems 48 Inc., Oakland, USA); in the P-ExPress group, keratometric parameters for calculation of the intraocular lens (IOL, SRK-T formula). The BCVA will be expressed as a logMAR unit; for example, a Snellen BCVA at 1.0 (100% or 20/20) equals a logMAR at 0.

Postoperative assessment was similar to that carried out before surgery, but particular attention was paid to possible complications after implantation, necessary additional procedures, and modification of glaucoma medication. Additional procedures were needle revision, 5-fluorouracyl (5-FU) subconjunctival injections (5 mg in 0.2 mL), and suture lysis. These were carried out if the following were present: elevated IOP (≥16 mmHg); the presence of subconjunctival fibrosis; undeveloped flat bleb. The development of subconjunctival fibrosis was diagnosed when engorged and tortuous conjunctival vessels over the flap were present. 5-FU injections were administered for 5 consecutive days or until fibrosis resolved and IOP stabilized provided that there were no side effects caused by the antimetabolites [8, 9]. Hypotony was defined as IOP ≤6 mmHg early at the first 7 postoperative days and late after 7 days after surgery.

Complete surgical success was defined as IOP ≤18 mmHg (exact value depends on target pressure) without the use of glaucoma medications; qualified success was defined as IOP ≤18 mmHg with a maximum of 2 glaucoma medications. IOP >18 mmHg with or without the usage of glaucoma medication and the need for revision surgery were considered unsuccessful.

*Statistical Evaluation*. In all groups, the arithmetic mean and standard deviation were calculated. Quantity and percentage distribution were calculated to determine qualitative characteristics. Data were tested for normality using the Shapiro-Wilk test. Between-group comparisons were made using Student's *t*-test. Within-group differences, obtained from data taken at different time points, were evaluated using paired Student's *t*-test and Wilcoxon signed-rank test for parametric and nonparametric data, respectively. *χ* ^2^ test of independence for two variables was used to compare quantitative characteristics. Values of *P* < 0.05 were considered statistically significant. Analyses were carried out using the SPSS and NCSS statistical packages.

## 3. Results

A total of 46 eyes were included in the P-ExPress group and 42 eyes were included in the ExPress group. The average follow-up time was 12.9 ± 0.4 months in the P-ExPress group (range: 9.4–13.5) and 12.2 ± 0.4 months in the ExPress group (range: 9.1–13.7).

### 3.1. Intraocular Pressure

The mean IOP before surgery in the P-ExPress group was 26.4 ± 9.3 mmHg and after 12 months it dropped by 29.7% to 18.8 ± 4.8 mmHg (*P* = 0.02). Over the same duration, mean IOP in the ExPress group decreased by 31.4% from 27.0 ± 10.9 mmHg to 18.1 ± 5.9 mmHg (*P* = 0.01). No significant differences between groups were found during the follow-up examinations (*P* > 0.05) (Table 2).

### 3.2. Glaucoma Medications

Over the 12-month evaluation period, the number of preoperative glaucoma medications in the P-ExPress group decreased from 3.3 ± 0.8 to 1.3 ± 1.7 (*P* = 0.023). In the ExPress group, glaucoma medications decreased from 2.9 ± 0.9 prior to surgery to 0.9 ± 1.65 over the same period (*P* = 0.019). No significant differences between the groups were found for the number of medications used pre- or postoperatively and at the end of observation period (*P* = 0.079). However, in the 6th month, the number of glaucoma medications was lower in the ExPress group (*P* = 0.003) (Table 3).

### 3.3. Surgical Success

**Figure 1:**
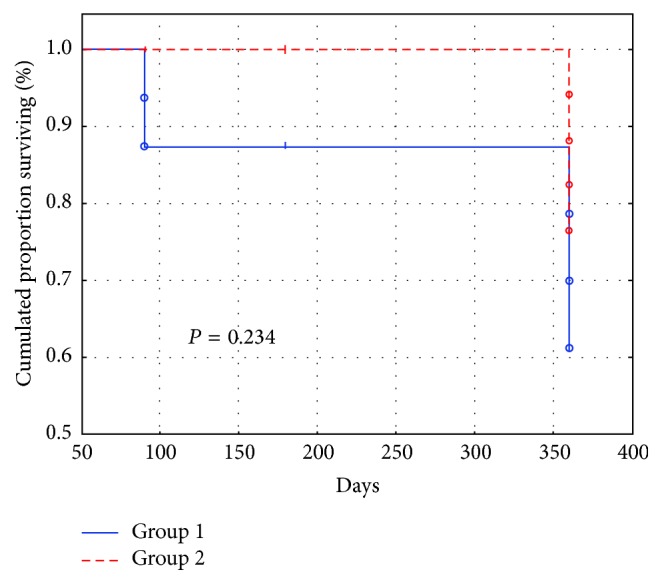
Cumulative surviving proportion (Kaplan-Meier) for success criterion of intraocular pressure less than or equal to 18 mmHg. Without medications, complete success rate.

No significant differences for complete or qualified success were found between the P-ExPress and ExPress groups (complete, 33.7% versus 41.3%, *P* = 0.23 (Figure 1); qualified, 61.4% versus 76.6%, *P* = 0.15).

### 3.4. Visual Acuity

Preoperatively, visual acuity in the P-ExPress group was 0.54 ± 0.56 logMAR and over the 12-month period it improved by 0.32 ± 0.49 logMAR (*P* = 0.001). In contrast, in the ExPress group, visual acuity remained relatively constant during the entire follow-up period: 0.33 ± 0.4 logMAR (preoperatively) and 0.37 ± 0.65 logMAR after 12 months (*P* = 0.95). BCVA values were significantly higher in the ExPress group, compared to the P-ExPress group, preoperatively (*P* = 0.02) and on the first postoperative day (*P* = 0.006) (Table 4). In 4 cases in P-ExPress group, the deterioration of vision occurred which was caused by secondary cataract. In these cases, the posterior YAG capsulotomy was performed.

### 3.5. Visual Field

Preoperatively, mean deviation (MD) on visual field testing was −17.62 ± 8.4 dB in the P-ExPress group and −14.4 ± 14.4 dB in the ExPress group (*P* = 0.12). After 12 months of observation, MD in the P-ExPress and ExPress groups was −13.2 ± 10.4 dB and −14.8 ± 8.9 dB (*P* = 0.35), respectively. Stabilization of the visual field was observed in 83.3% of patients from the P-ExPress group and in 89.7% of patients from the ExPress group (*P* = 0.14). Improved MD was found in 16.7% of patients in the P-ExPress group and in 10.3% of patients in the ExPress group (*P* = 0.24).

The profile of postoperative complications is shown in Table 5.

5-FU subconjunctival injections were administered to 13 patients from the P-ExPress group (28.2%) and 11 patients from the ExPress group (26.1%) (*P* = 0.87). Needling was required in 21 ExPress inserts from the P-ExPress group (16.2%) and in 18 inserts from the ExPress group (15.5%) (*P* = 0.87). A single patient from the P-ExPress group (2.1%) and 4 patients from the ExPress group (11.1%) underwent laser suture lysis (*P* = 0.14). An additional sealing suture was used in 1 patient from the P-ExPress group (0.78%) (*P* = 0.342). Reoperation was necessary in 2 subjects from the P-ExPress group due to extrusion of the drainage implant through the scleral flap and fibrosis of the filtration bleb. In both cases, classical trabeculectomy was carried out. Postoperative (but not preoperative) data from these patients were excluded from analyses.

## 4. Discussion

In our study, the average age of patients from the P-ExPress group was higher than that from the ExPress group. This is most likely due to the association between cataract development and old age.

Preoperative IOP values did not differ between the P-ExPress and ExPress groups, although during the early postoperative phase (days 1, 7, and 30) IOP was significantly higher in the P-ExPress group. The risk of increased intraocular pressure immediately after phacoemulsification is well documented and it tends to be temporary [10–12]. Włodarczyk et al. [13] examined the effect of phacoemulsification on IOP and noted a 3.3 mmHg increase during the first day after this procedure. In 10% of cases, the increase exceeded 23 mmHg. After 7 days, the authors noted a 2.9 mmHg drop in IOP, which was a relative decrease of 2.1 mmHg compared to preoperative values.

Several factors may account for the increase in IOP, most often occurring between the 6th hour and the 8th hour after the procedure. These include incomplete rinsing of viscoelastic materials from the anterior chamber, local inflammation caused by cortical or lens fragments, blood cells, fibrin or free radicals, damaged aqueous veins, and blockage of pupil caused by mydriasis [14, 15]. In glaucomatous eyes, an additional factor is the increased resistance to aqueous outflow; thus dysregulation is more common and may contribute to the raised IOP. Although IOP usually normalizes after treatment, elevated IOP may cause permanent damage to the visual field, especially among patients with residual changes. For this reason, all possible precautions should be undertaken to minimize risk, such as complete removal of cortical debris/viscoelastic material and limited contact of surgical apparatus with the iris [10, 11]. In our study, increased IOP was observed in 7 patients in the P-ExPress group (16%) and developed over the first 24 hours after surgery, with a maximum value of 27 mmHg. In all cases, IOP returned to normal levels within 72 hours.

Many studies have focused on the long-term influence of cataract surgery on IOP [16]. It has been observed that in patients with open-angle glaucoma IOP dropped following phacoemulsification and remained almost stable for 12–48 months [17]. A number of studies have since confirmed this finding and observed drops in IOP from 1.4–1.9 mmHg to 4.9–5.3 mmHg [12, 14, 18, 19]. Three potential mechanisms may be responsible for the change in IOP following phacoemulsification: (1) decreased production of the aqueous humor as a result of pulling the vitreous through the fibers of the ciliary body, which is caused by the contraction of the lens capsule; (2) improved aqueous outflow through the trabecular meshwork and Schlemm's canal; (3) improved corneoscleral outflow [19]. The latter two mechanisms seem most probable and are consistent with the results of Meyer et al. [14] who demonstrated that improved aqueous outflow in glaucoma patients, following phacoemulsification, is a consequence of prostaglandin synthesis and release during surgery. The precise mechanism is still unclear; however, it may involve PGE-1, which increases the aqueous outflow, or an alternative pathway involving PGF-2 [20, 21]. Salim [1] reviewed the clinical findings from studies using the ExPress mini implants and noted that the decreased IOP is comparable to the gold standard, trabeculectomy, and underlined the fact that there was reduced incidence of intra- and postoperative complications. Furthermore, ExPress implants are also an effective treatment for eyes with advanced glaucoma damage where it is essential to keep the IOP as low as possible [22].

Given that phacoemulsification can lower IOP, in this study we tested the hypothesis whether phacoemulsification combined with ExPress implantation would have greater ocular hypotensive effects. Surprisingly, we found that P-ExPress and ExPress groups did not differ in their mean IOP during the short-term postoperative period (3–12 months). However, similar findings were reported by Kanner et al. [23] who compared the effectiveness of ExPress implants alone and combined with phacoemulsification (P-ExPress). In their study, a total of 345 eyes were examined, 231 were treated with ExPress implants, and 144 eyes received the combined treatment. A 3-year follow-up revealed that baseline IOP in the ExPress group was 27.9 ± 10.7 mmHg, whereas in the P-ExPress group pressure was 20.9 ± 7.9 mmHg. During the early postoperative period, the reduction in IOP was significantly higher in ExPress group compared to the P-ExPress group (8.7 ± 16.6 and 2.3 ± 11.3 mmHg; postoperative day 1). The authors suggested that the initial differences in IOP resulted from usage of viscoelastic materials in the combined treatment. At later time points, reductions in IOP were observed in both groups. One year following surgery the mean decrease in IOP was found to be 13.5 ± 6.1 and 15.1 ± 8.6 mmHg in the ExPress and P-ExPress groups, respectively. The mean decrease 3 years after surgery was 16.4 ± 4.1 and 16.8 ± 5.1 mmHg in the ExPress and P-ExPress groups, respectively. In line with our study, the mean age of patients in the P-ExPress group was significantly higher than that of patients in the ExPress group and BCVA at baseline was lower.

In both groups, the number of glaucoma medications was reduced postoperatively. However, at the 12th month after surgery, the number of medications was marginally higher in the P-ExPress group, compared to the ExPress group, although this did not reach statistical significance. This is in line with reports showing that phacotrabeculectomy and combined phacoemulsification with ExPress implantation are less effective strategies to control IOP compared to ExPress implantation or trabeculectomy alone [24, 25]. In our study, we observed a trend for success rate (complete and qualified) to be lower in the P-ExPress group compared to the ExPress group, although this difference did not reach statistical significance.

In our study, a greater number of subconjunctival 5-FU injections were required in the P-ExPress group (28.2% versus 26.1%) due to increased fibrosis of the filtration bleb. This group experienced more often fibrinous exudation in the anterior chamber, most likely the result of increased release of inflammatory mediators during the phacoemulsification [26].

In a multicentre randomized study of 120 patients, Netland et al. compared the findings of patients implanted with ExPress mini-devices (*n* = 59) with conventional trabeculectomy (*n* = 61). The mean IOP values at baseline were comparable in both groups (25.1 ± 6.0 mmHg versus 26.4 ± 6.9 mmHg). Within 6 months following surgery, IOP was lower in patients from the trabeculectomy group (13.8 ± 4.7 mmHg versus 11.9 ± 4.6 mmHg). At 2 years, IOP was equivalent in both groups (14.7 ± 4.6 mmHg in ExPress group and 14.6 ± 7.1 mmHg in trabeculectomy group). Complications occurred in 18.6% of cases treated with ExPress device and in 41% of cases that underwent trabeculectomy. In our study, the mean IOP (15.31 ± 2.8 mmHg) in the ExPress group was similar to that reported by Netland et al. [27]. We found more complications in the group that underwent combined surgery (P-ExPress) compared to the implantation alone (ExPress).

Possible visual impairment following glaucoma and cataract surgery presents an important clinical challenge. Cataract surgery may deteriorate the function of the filtration bleb in patients after glaucoma procedure [28]. It has been suggested that mini-device implantation in phakic eyes may accelerate cataract development [29]. Our study did not find any postoperative differences in BCVA in the ExPress group versus P-ExPress group regardless of the phase of observation. Visual acuity in the P-ExPress group improved compared to baseline but did not differ from the ExPress group. No cataractogenic effect of the standard ExPress implant was observed, although the period of observation was much shorter compared to Jampel's study (12 versus 36 months).

There is much debate about the outcome of combined versus sequential surgery for the management of coexisting cataract and glaucoma. Performing cataract extraction and phacoemulsification is an attractive proposition because it saves time and costs and reduces patients' stress. It is also reasonable to assume, as proposed by Shields [30] and later by Brown et al. [31], that in patients with a stable IOP the first-choice surgical procedure should be removing the cataract, but combined procedure is recommended for patients with borderline IOP. A sequential procedure is recommended in cases where the IOP is high.

## 5. Conclusions

ExPress implants alone or combined with phacoemulsification are safe and effective surgical treatments for open-angle glaucoma. Coexistence of cataracts does not constitute a compelling contraindication for combined surgery.

## Figures and Tables

**Table 1:** Patient's demographic data.

Group	P-ExPress	ExPress	*P*
Follow-up (months)	12.9 ± 0.4	12.2 ± 0.6	0.896^1^
*n*	46	42	0.764^1^
Age (years)	71.8 ± 9.46	66.1 ± 10.83	0.021^1^
Sex (female/male)	29/17	18/24	0.047^2^
Eye (right/left)	25/21	14/28	0.037^2^

Glaucoma type			
POAG	29	33	
PEX	15	7	>0.05^2^
Pigmentary	2	2	

^1^Student's *t*-test; ^2^ *χ* ^2^ test.

**Table 2:** Intraocular pressure (IOP) mean values, median values, standard deviations, and range in the phaco-ExPress (P-ExPress) and ExPress (ExPress) groups at specific times after surgery.

Time	P-ExPress			ExPress			*P*
	Mean (SD)	Median	Range	Mean (SD)	Median	Range	
Pre-op	26.4 ± 9.3	25.00	20–30	27.0 ± 10.9	24.00	19–30	0.877
1st day	17.2 ± 9.0	16.00	11–21	15.3 ± 6.3	15.00	12–18	0.411
7th day	16.1 ± 7.6	14.00	11–21	15.2 ± 6.0	15.00	10–19	0.869
1st month	17.8 ± 11.8	16.00	13–20	15.4 ± 4.1	16.00	13–18	0.582
3rd month	15.0 ± 4.1	16.00	13–17	15.7 ± 2.6	16.00	14–18	0.427
6th month	15.0 ± 3.5	15.50	12–28	15.3 ± 2.9	16.00	14–17	0.733

9th month	15.4 ± 5.6	16.00	11–18	15.5 ± 3.2	17.00	16–18	0.249
12th month	18.8 ± 4.8	18.50	15–22	18.1 ± 5.9	18.00	16–18	0.814

Student's *t*-test.

**Table 3:** Amount of hypotensive drugs: mean values, median values, standard deviations, and range in the phaco-ExPress (P-ExPress) and ExPress alone (ExPress) groups at specific times after surgery.

Time	P-ExPress group		ExPress group		*P*
	Mean	Median	Mean	Median	
Pre-op	3.3 ± 0.8	3.0	2.9 ± 0.9	3.0	0.079
7th day	0.1 ± 0.3	0.0	0.1 ± 0.3	0.0	0.968
1st month	0.1 ± 0.4	0.0	0.2 ± 0.5	0.0	0.535
3rd month	0.4 ± 0.7	0.0	0.3 ± 0.6	0.0	0.585
6th month	1.4 ± 1.2	2.0	0.5 ± 1.0	0.0	0.003

9th month	1.4 ± 1.0	2.0	1.0 ± 1.6	0.0	0.177
12th month	1.3 ± 1.7	0.0	0.9 ± 1.65	1.0	0.419

Mann-Whitney *U* test.

**Table 4:** Visual acuity (logMAR) mean values, median values, standard deviations, and range in the phaco-ExPress (P-ExPress) and ExPress (ExPress) groups at specific times after surgery.

Time	P-ExPress			ExPress			*P*
	Mean (SD)	Median	Range	Mean (SD)	Median	Range	
Pre-op	0.54 ± 0.56	0.3	0.16–0.77	0.33 ± 0.4	0.16	0.0–523	0.020
1st day	0.8 ± 0.57	0.7	0.3–1.22	0.45 ± 0.36	0.3	0.16–0.67	0.006
7th day	0.5 ± 0.65	0.22	0.97–0.67	0.37 ± 0.33	0.3	0.097–0.52	0.968
1st month	0.41 ± 0.5	0.22	0.97–0.67	0.27 ± 0.33	0.16	0.024–0.398	0.444
3rd month	0.24 ± 0.44	0.13	0.0–0.22	0.34 ± 0.5	0.1	0.0–0.4	0.674
6th month	0.24 ± 0.45	0.05	0.0–0.19	0.24 ± 0.36	0.16	0.0–0.301	0.378

9th month	0.42 ± 0.61	0.22	0.0–0.398	0.29 ± 0.46	0.05	0.0–0.398	0.687
12th month	0.32 ± 0.49	0.19	0.0–0.467	0.37 ± 0.67	0.1	0.0–0.650	0.829

Mann-Whitney *U* test.

**Table 5:** Postoperative complications.

Complications	P-ExPress *n* (%)	ExPress *n* (%)	*P* ^*∗*^
Intraoperative			
Bleeding	—	—	—
Postoperative	—	—	—
Hyphema			
Blood level in AC	*1 (2.1)*	—	—
Erythrocytes in AC	—	—	—
Wound leakage	*1 (6.5)*	—	—
Fibrosis	*7 (15.2)*	*5 (11.9)*	*0.871*
Anterior chamber cells	*3 (6.5)*	—	—
Hypotony			
Until 7 days	—	*2 (4.8)*	—
Until 30 days	—	*1 (2.4)*	—
Until 180 days	—	—	—
Choroid detachment	—	*1 (2.4)*	—

^*∗*^ *χ* ^2^ test.
